# Immunotherapy for people with clinically isolated syndrome or relapsing-remitting multiple sclerosis: treatment response by demographic, clinical, and biomarker subgroups (PROMISE)—a systematic review protocol

**DOI:** 10.1186/s13643-022-01997-2

**Published:** 2022-07-01

**Authors:** Thomas Lehnert, Christian Röver, Sascha Köpke, Jordi Rio, Declan Chard, Andrea V. Fittipaldo, Tim Friede, Christoph Heesen, Anne C. Rahn

**Affiliations:** 1 Institute of Neuroimmunology and Multiple Sclerosis, University Medical Center Hamburg-Eppendorf (UKE), Hamburg, Germany; 2 Department of Medical Statistics, University Medical Center Göttingen, Göttingen, Germany; 3 Institute of Nursing Science, Faculty of Medicine and University Hospital Cologne, University of Cologne, Cologne, Germany; 4 Neurology/Neuroimmunology, Centre d’Esclerosi Multiple de Catalunya (Cemcat), Hospital Universitari Vall d’Hebron, Universitat Autonoma de Barcelona, Barcelona, Spain; 5 Department of Neuroinflammation, UCL Queen Square Institute of Neurology, Faculty of Brain Sciences, University College London, London, UK; 6 National Institute for Health Research (NIHR), University College London Hospitals (UCLH) Biomedical Research Centre, London, UK; 7 Department of Oncology, Istituto Ricerche Farmacologiche “Mario Negri” IRCCS, Milano, Italy; 8 Institute for Social Medicine and Epidemiology, Nursing Research Unit, University of Lübeck, Lübeck, Germany

**Keywords:** Multiple sclerosis, Systematic review, Meta-analysis, Immunotherapy, Treatment response, Subgroups

## Abstract

**Background:**

Multiple sclerosis (MS) is an inflammatory and degenerative disease of the central nervous system with an increasing worldwide prevalence. Since 1993, more than 15 disease-modifying immunotherapies (DMTs) have been licenced and have shown moderate efficacy in clinical trials. Based on the heterogeneity of the disease and the partial effectiveness of therapies, a personalised medicine approach would be valuable taking individual prognosis and suitability of a chosen therapy into account to gain the best possible treatment effect.

The primary objective of this review is to assess the differential treatment effects of all approved DMTs in subgroups of adults with clinically isolated syndrome or relapsing forms of MS. We will analyse possible treatment effect modifiers (TEM) defined by baseline demographic characteristics (gender, age), and diagnostic (i.e. MRI measures) and clinical (i.e. relapses, disability level) measures of MS disease activity.

**Methods:**

We will include all published and accessible unpublished primary and secondary analyses of randomised controlled trials (RCTs) with a follow-up of at least 12 months investigating the efficacy of at least one approved DMT, with placebo or other approved DMTs as control intervention(s) in subgroups of trial participants. As the primary outcome, we will address disability as defined by the Expanded Disability Status Scale or multiple sclerosis functional composite scores followed by relapse frequency, quality of life measures, and side effects. MRI data will be analysed as secondary outcomes.

MEDLINE, EMBASE, CINAHL, LILACS, CENTRAL and major trial registers will be searched for suitable studies. Titles and abstracts and full texts will be screened by two persons independently using Covidence. The risk of bias will be analysed based on the Cochrane “Risk of Bias 2” tool, and the certainty of evidence will be assessed using GRADE.

Treatment effects will be reported as rate ratio or odds ratio. Primary analyses will follow the intention-to-treat principle. Meta-analyses will be carried out using random-effects models.

**Discussion:**

Given that individual patient data from clinical studies are often not available, the review will allow to analyse the evidence on TEM in MS immunotherapy and thus support clinical decision making in individual cases.

**Systematic review registration:**

PROSPERO CRD42021279665.

**Supplementary Information:**

The online version contains supplementary material available at 10.1186/s13643-022-01997-2.

## Background

Multiple sclerosis (MS) is a chronic disease of the central nervous system, characterised by the destruction of myelin and axons, associated with focal inflammation and diffuse neurodegeneration in the brain as well as spinal cord grey and white matter [1]. Disease onset is typically between the ages of 20–40 [2]. With a global median prevalence of 33 per 100,000 people and approximately 2.5 million people affected worldwide, MS is the most common neuroimmunological disorder and most frequent cause of non-traumatic disability in young adults [3, 4]. While the pathogenesis of MS is not completely understood, current evidence suggests that a multifactorial interplay of genetic, environmental and lifestyle risk factors results in a dysregulated immune response [5, 6]. The prognosis and course of the disease are highly variable and currently unpredictable at the individual patient level, particularly at disease onset [7, 8]. MS negatively impacts many health-related outcomes, such as activities of daily living [9], quality of life [10, 11] and mortality [12], and places a large financial burden on patients and their families, healthcare systems and societies at large [13, 14].

In most people, MS is relapsing at the beginning (relapsing-remitting MS (RRMS)), with exacerbations followed by partial or full remission of neurological symptoms and phases of relative stability [2, 15]. As evidence has shown that neurodegeneration is present from the earliest stages of MS [16], these clinical subtypes should be considered as different points on a continuum, with focal white matter inflammation being dominant during the relapsing-remitting phase and neurodegeneration during the progressive phase [2, 17, 18]. Thus, neurodegeneration is considered the main driver of disability progression [19, 20].

Over the last three decades, more than 15 immunotherapies (disease-modifying therapies (DMTs)) with different mechanisms of action and profiles of benefits and risks have been approved for the treatment of relapsing MS [21, 22]. Primarily targeting the focal inflammatory processes of the disease, these treatments reduce brain white matter lesion formation, as seen using Magnetic resonance imaging (MRI), reduce relapse rates and possibly slow disability accrual and overall disease progression [23–25]. However, their long-term benefit is still uncertain [26–28]. Evidence from RCTs and observational studies suggests that starting a DMT soon after diagnosis potentially leads to better (long-term) outcomes in persons with RRMS, such as a reduced risk of relapses and transition to SPMS, when compared to later initiation [29–32]. This favourable effect seems to be more pronounced for DMTs with higher efficacy as alemtuzumab, fingolimod, ocrelizumab, ozanimod, and natalizumab [33, 34]. DMTs with higher efficacy also exhibit higher toxicity [24], which in rare cases may lead to severe and potentially life-threatening adverse effects, e.g. progressive multifocal leukoencephalopathy (PML) [35, 36].

The side-effect profile of a DMT affects the adherence to the DMT [37]. While treatment adherence is essential to the effectiveness of DMTs [38], non-adherence was reported in up to 70% of people using DMTs [39, 40]. Consequently, a substantial group of DMT users may not experience the full benefits, thus having an increased risk of new or enlarging brain lesions, relapses and worsening disability [41, 42].

To improve care including immunotherapy management, the concept of personalised (precision) medicine is highly relevant in MS. However, the implementation of such an approach in MS is currently limited by knowledge gaps. The pathogenesis of MS is not fully understood. Robust and validated biomarkers and validated treatment algorithms are lacking [43]. Hence, the current approach to personalised immunotherapy in MS depends on an evidence-based estimate of the prognosis, a subsequent choice of therapy based on a shared decision-making (SDM) approach and the evaluation of early immunotherapy responses [44].

Against the background of the heterogeneous clinical presentation, progressive nature of MS and DMTs that are only partially effective in controlling neurodegeneration [19], it is crucial to optimise the use of available DMTs during the early inflammatory phase of the disease to prevent the accumulation of irreversible neurological damage and concomitant accrual of disability [45]. The optimal treatment window may close early in the disease course, i.e. before the walking distance is impaired [34, 46]. Therefore, the reliable identification of patients with a poor response is crucial [25, 47]. One method to identify poor responders lies in the assessment of treatment effect modifiers (TEMs) in RCTs [47, 48], which are baseline variables associated with larger or smaller treatment effects [47, 49]. TEMs are defined in relation to a specific treatment and against a control group and are typically assessed via subgroup analysis in RCTs [49]. The clinical rationale for the assessment of TEM is to ascertain whether the effect of a DMT differs in groups of patients with different baseline characteristics [50]. Thus, TEM can help to identify groups of patients who have lower than average benefits from a DMT (poor responders) or, at worst, no benefit at all (non-responders), but also patients who benefit above-average from a treatment.

To our knowledge, there is no systematic review considering all presently available DMT options for all people with clinically isolated syndrome (CIS) or RRMS [51–54].

We will consider all DMTs currently approved by the European Medicines Agency (EMA) or the U.S. Food and Drugs (FDA) for use in people with CIS or RRMS as alemtuzumab, azathioprine, beta interferons (interferon beta-1a, interferon beta-1b), cladribine, dimethyl, fingolimod, glatiramer acetate, mitoxantrone, natalizumab, ofatumumab, ozanimod, ocrelizumab, ponesimod, siponimod and teriflunomide. A key goal of current research is precision medicine, which is tailoring treatment to individuals rather than the blanket treatment of groups. For people with MS, we now have a large range of treatments; some with similar degrees of efficacy when assessed at a group level. At an individual level, treatment effects are not homogeneous across trial participants as suggested by findings from subgroup analyses of individual RCTs [55, 56] but vary according to demographic and disease-related characteristics [51, 53, 57, 58]. Evidence indicates that higher treatment benefit is associated with baseline characteristics of earlier (i.e. younger age, lower levels of disability) and more inflammatory disease activity as shown on MRI [51, 57, 58]. In the following, we outline major possible TEMs, i.e. age, disability level, sex, relapse rate, MRI activity and previous DMT use (all assessed at baseline).

### Age

In MS, the risk of a progressive disease course increases with age [59]. Neuropathological evidence indicates that earlier inflammation, while still present, is more compartmentalised and diffuse later compared to early disease stages [60]. In addition, neurodegeneration is more apparent and may increasingly become the dominating factor determining clinical outcomes. These processes most likely evolve rather than simply progress on a continuum from relapsing-remitting to secondary progressive MS [59]. Further, ageing of the immune system leads to a decline in the regulation of innate and as well as adaptive immune responses [58, 61], possibly also altering responses to DMTs. A recent meta-analysis of 38 clinical trials that assessed the efficacy of DMTs on disability progression found age to be an essential modifier of DMT efficacy [58]. The authors concluded that higher efficacy treatments exert their benefit over lower efficacy treatments only during the early stages of MS, and beyond the age of 53 years, the model suggests that for the average person with MS, DMTs have no added value. Similarly, a meta-analysis of six RCTs by Signori and colleagues [51] found that treatment effects on the annualised relapse rate (ARR) were significantly higher in participants who were <40 years of age compared to those ≥40 years.

### Disability level

Disability, as measured by the Expanded Disability Status Scale (EDSS), correlates with disease duration, albeit with substantial individual variation in longer-term clinical outcomes. Beyond an EDSS value of 4.0, MS is increasingly likely to be running a progressive rather than relapsing-remitting course [46]. It is well-recognised that many MS treatments have no significant effects on disease progression (at least as assessed using EDSS) without relapses [62]. Since it may take several years for a progressive MS course to develop and manifest itself, EDSS may be a more timely, if imperfect, proxy for this. However, to date, this has not thoroughly been looked at. The multiple sclerosis functional composite (MSFC) represents an integrated disability measure possibly more sensitive to change than the EDSS [63, 64]. While the mobility-dependent EDSS shows floor effects at higher disability levels [65], the MSFC and especially hand function [66] and information processing measures may show more room for improvement. On the other hand, substantially impaired information processing may indicate irreversible neurodegeneration [67] and thereby lowering chances for a treatment benefit. Therefore, the impact of these measures on treatment responses is meaningful.

### Sex

MS is increasingly prevalent among women, but clinically men are more likely to develop a progressive course [15] and do so faster (both progression to SPMS from disease onset and age at SPMS diagnosis) [68]. Sex differences in immunological, neurodegenerative, and neuroprotective mechanisms, which are increasingly found in healthy individuals may also be seen in MS [69, 70]. While sex hormones and their dynamic interplay seem to be major drivers, other x-chromosomal factors might be also relevant. Women seem to have stronger inflammatory responses, but also a greater capacity for repair in the CNS [71]. For these reasons, it seems likely that responses to DMTs differ between females and males. However, so far, no clear patterns for the effects of sex on immunotherapy efficacy have been reported [57].

### Relapse rate

Relapse rates are typically the main factor determining eligibility for treatments in clinical practice and entry to clinical trials and are the primary outcome of most RRMS treatment studies. One to two relapses in the preceding years are an inclusion criterion in almost every RRMS treatment trial, as there needs to be sufficient disease activity in the comparator arms for a treatment effect to be demonstrable. Relapse activity correlates with MRI inflammatory disease activity and relapse-associated disability accrual. The presence of superimposed relapses and/or MRI inflammatory activity also appears to predict treatment response in a subset of people with progressive MS [72], albeit when using treatments that typically have also been shown to be effective in RRMS, rather than agents that may target non-inflammatory processes. Overall, relapse rates have only a minor impact on disability evolution [73]. While in the earliest treatment trials in MS, participants had substantially higher relapse rates prior to study inclusion [74] than in more recent studies [75], absolute relapse rate reduction and percentages of relapse-free patients do not differ too much (15 versus 11 out of 100). Relapse rate before treatment initiation might have only a minor influence on subsequent relapse activity under treatment but may represent a TEM for the progression of disability.

### MRI activity

MRI is a highly established diagnostic tool in MS [76]. However, its prognostic value is a matter of ongoing debate. A high number of T2 lesions at presentation as well as an early increase of T2 lesion number has been regarded as an indicator of a greater risk of disability progression [77]. Recent data underline the importance of lesion location, i.e. early infratentorial and spinal cord lesions as possibly more predictive for later progression [7, 78]. As all DMTs impact on lesion evolution, a treatment effect on MRI-visible lesion activity is (at a group level) a good predictor of clinical treatment efficacy [8]. Slowed lesion accumulation moreover correlates with disability progression in the short term [79], albeit this association is not without criticism [80]. Although the long-term predictive value of MRI measures is not clear, some studies have hypothesised that the accumulation of new active lesions in the first stages of therapy may impact long-term disability [81, 82].

### Previous DMT use

Breakthrough disease on a low to moderate immunotherapy may indicate another level of activity of the immune processes. In clinical practice, DMTs are often applied as escalation therapies in case of previous treatment failure. Very few studies have focussed on non-responders, though, such as CARE-MS2 investigating alemtuzumab [83]. The pressing clinical question, if pretreatment or previous treatment failure reduces the chances of a treatment benefit, remains unanswered. While this has not been thoroughly addressed, registry data indicate that the probability of converting to SPMS is significantly lower for patients escalated from glatiramer acetate or interferon beta to fingolimod, alemtuzumab or natalizumab within 5 years of disease onset compared with matched patients who were escalated later [29]. Therefore, the sequence of treatments might also be predictive for treatment response, both in terms of relapses and disability progression.

In summary, the identification of baseline factors consistently associated with larger treatment effects (response) can help to optimise MS immunotreatment by channelling people with MS towards the most effective DMT, thus not exposing them to ineffective and potentially harmful treatments. This may in turn reduce non-adherence and premature treatment termination. Identifying and quantifying the effect baseline TEMs should result in a more nuanced understanding of the effect of a DMT and will help to set the overall effects of DMTs into context [84, 85]. Identification of factors predicting higher or lower than average response to specific DMTs at treatment initiation can inform clinical treatment guidelines and ultimately contribute towards personalised treatment in people with MS [86].

## Methods

The objective of this review is to assess differential treatment effects (response) of approved immunotherapies in subgroups of adults with CIS or RRMS, defined by baseline age, sex, treatment history, relapse rate, disability level, and MRI activity.

### Criteria for considering studies for this review

#### Types of studies

We will include all published and unpublished primary and secondary analyses of RCTs including randomised crossover trials with a follow-up of at least 12 months, in which the efficacy of at least one approved DMT, with placebo or other approved DMTs as control intervention(s), was investigated in subgroups of trial participants. Subgroup characteristics need to be measured at baseline, i.e. prior to randomisation before participants were exposed to the study drug. We will consider subgroups defined by age, disability level, sex, relapse history, MRI activity and treatment history, which are described and operationalised under Data Synthesis below. We will include studies regardless of publication status and language.

#### Types of participants

We will include participants aged ≥18 years with CIS or RRMS, according to established diagnostic criteria, as applied in the original studies, and treated with FDA or EMA approved DMTs. CIS is defined as a first monophasic clinical episode typical of CNS demyelination suggestive of MS, while para-clinical findings do not justify a diagnosis of MS [87, 88]. Definition of RRMS has to be based on a diagnosis according to established diagnostic criteria, i.e. Schumacher [89], Poser [90], original 2001 McDonald [91] or modified McDonald [92, 93] criteria. We will exclude studies on people with progressive forms of MS [87]. In the case of trials quoting results for mixed populations of participants with relapsing as well as progressive forms of MS, they will be included if more than 75% of participants in the study had relapsing MS.

#### Types of interventions

We will include studies that evaluated one or more of the following DMT regimens as monotherapy:

- Alemtuzumab (Lemtrada®)
- Azathioprine (e.g. Imurek®)
- Cladribine (Mavenclad®)
- Dimethyl fumarate (Tecfidera®)
- Fingolimod (Gilenya®)
- Glatiramer acetate (Copaxone®, Clift®)
- Interferon beta-1a (Rebif®, Avonex®), pegylated interferon beta-1a (Plegridy®)
- Interferon beta-1b (Betaferon®, Betaseron®, Extavia®)
- Mitoxantrone (Novantron®, Ralenova®)
- Natalizumab (Tysabri®)
- Ofatumumab (Kesimpta®)
- Ozanimod (Zeposia®)
- Ocrelizumab (Ocrevus®)
- Ponesimode (Ponvory®)
- Siponimod (Mayzent®)
- Teriflunomide (Aubagio®)

Comparators will be placebo or other approved DMTs.

#### Types of outcome measures

The selection and classification of outcomes were conducted in a structured process among the review authors and two patient representatives. Initially, a list of treatment outcome domains used in studies on people with MS was compiled based on expert knowledge and published literature [94–96]. This list was adapted and reduced in two rounds of structured group discussions with two patient representatives from the German Multiple Sclerosis Society [97]. In a final step, the representatives were asked to classify outcomes as critical outcomes and important outcomes.

#### Primary outcomes

##### Critical outcomes

- Disability worsening: number or proportion of participants experiencing, or free from disability-worsening at 12, 24, 36 or 48 months after randomisation, yearly until the end of follow-up (if longer than 48 months) or at the end of follow-up. Disability worsening must have been assessed using either the Expanded Disability Status Scale (EDSS) [98] or the multiple sclerosis functional composite (MSFC) [63]. The EDSS is a method used to quantify disability in MS based on a neurological examination [98]. The MSFC assesses disability with three different tests: walking ability (25 foot walk test), hand functioning (9 hole peg test) and information processing (symbol digit modalities test) [63]. The MSFC can produce scores for each of the three individual measures as well as a composite score, which is a continuous variable that can be used as any numerical variable in analyses. MSFC disability worsening is defined as an increase in scores in at least one MSFC component by 20% from baseline, sustained for at least 3 months [99].
- Relapses: number of relapses, ARR or proportion of participants with new relapses at 12, 24, 36 or 48 months after randomisation, yearly until the end of follow-up (if longer than 48 months) or at the end of follow-up. While a relapse is typically defined as one or more newly developing or reoccurring symptoms of neurological dysfunction persisting for more than 24 h, preceded by a period of stability for at least 1 month [91], other relapse definitions will be accepted as well.
- Quality of life (QoL): questionnaires assessing generic or MS-specific QoL (e.g. Leeds Multiple Sclerosis Quality of Life (LMSQoL), Functional Assessment of Multiple Sclerosis (FAMS), Hamburg Quality of Life Questionnaire in Multiple Sclerosis (HAQUAMS), Multiple Sclerosis International Quality of Life (MUSIQoL) questionnaire, Multiple Sclerosis Quality of Life-54 (MSQoL-54), Health Status Questionnaire 36 (SF-36) and EuroQol-5 Dimension (EQ-5D).
- Adverse events (AE): total number of serious adverse events or number of participants with at least one serious adverse event at the end of follow-up.

#### Secondary outcomes

##### Important outcomes

- MRI activity: The number or proportion of participants with new/enlarging T2 lesions or new T1 contrast-enhancing lesions at 12, 24, 36 or 48 months after randomisation, yearly until the end of follow-up (if longer than 48 months) or at the end of follow-up.
- No Evidence of Disease Activity (NEDA): number or proportion of participants achieving NEDA-3 at 12, 24, 36 or 48 months after randomisation, yearly until the end of follow-up (if longer than 48 months) or at the end of follow-up months. NEDA-3 is defined as an absence of MRI activity (i.e. new or enlarging lesions), relapses and disability worsening (as measured by EDSS) [100].
- Activities of daily living (ADLs): questionnaires assessing basic or instrumental ADLs or both (e.g. Functional Independence Measure (FIM), Katz ADL Index, Barthel Index).
- Fatigue: questionnaires assessing fatigue (e.g. Fatigue Severity Scale (FFS), Modified Fatigue Impact Scale (MFIS), Multidimensional Fatigue Index (MFI), and visual analogue scale (VAS) for fatigue).
- Conversion to clinically definite MS: time to conversion to clinically definite MS.
- Conversion to SPMS: time to conversion to SPMS [87].

### Search methods for identification of studies

We will not apply any language restrictions to the searches.

#### Electronic searches

We will search the following sources for eligible reports in any language:

- Cochrane Central Register of Controlled Trials (CENTRAL 2020) in the Cochrane Library
- MEDLINE (PubMed) (1946 to search date)
- Embase (Embase.com) (1974 to search date)
- Cumulative Index to Nursing and Allied Health Literature (CINAHL) (EBSCO host) (1981 to search date)
- Latin American and Caribbean Health Science Information Database (LILACS) (Bireme) (1982 to search date)
- ClinicalTrials.gov (http://clinicaltrials.gov)
- World Health Organization (WHO) International Clinical Trials Registry Platform (https://trialsearch.who.int/)

The search keywords are listed in Additional file 1.

#### Searching other resources

In addition to the electronic searches, we will screen the reference lists of published reviews and of included full-text articles for additional references to identify relevant trials.

### Data collection and analysis

#### Selection of studies

Data management and extraction will be conducted following the methods described in the *Cochrane Handbook for Systematic Reviews of Interventions* [101]. To select search titles and abstracts considered relevant for the review, two review authors will independently assess the results of the various searches using Covidence. Disagreements will be resolved by discussion with a third team member. Should more information be required to determine eligibility, we will contact study authors for additional information and check related publications.

Multiple reports of the same study will be collated. All potentially relevant reports will be retrieved in full-text and investigated further for inclusion independently by two authors. We will resolve disagreements through discussion and/or consensus with another author. To assure a reproducible process, we will screen full texts following the standardised inclusion and exclusion criteria. Reasons for study exclusion will be recorded and reported in detail in the “Characteristics of excluded studies” table and the process of study selection will be documented in a flow chart according to PRISMA guidelines [102].

#### Data extraction and management

Data extraction from included studies will be performed by two authors independently using a pre-defined data extraction form within an Excel spreadsheet. In case of disagreement not resolved by discussion, we will consult another review author for clarification; if not possible, the record will be assigned “awaiting assessment” and study authors will be contacted for clarification. The data extraction sheet will be designed to extract study-level and subgroup-level data, i.e. for the subgroups which this review aims to summarise: age, sex, treatment history, relapse rate, disability level and MRI activity (see “Data synthesis” section). The data extraction sheet will be piloted and assessed for usability within the review team. Study names and authors will not be masked. The following information will be extracted from individual studies:

- Study details: name/acronym, registration number(s), date, country, reports, and authors
- Eligibility criteria (inclusion/exclusion)
- Study design and clinical setting (e.g. mono or multi-centric)
- Number and characteristics of participants in the overall trial and each treatment arm (e.g. age, gender, years since diagnosis, MS type, level of disability) and number of participants in each treatment arm of the subgroups to be summarised in this review
- Intervention details: description of the intervention(s), dosage, regimen, frequency, duration
- Subgroup details (for all subgroups reported in a study): rationale for inclusion, definition, operationalisation (e.g. categories, thresholds), statistical analyses
- Length and description of participant follow-up
- Data analysis: statistical models used to conduct subgroup analysis, adjustment: co-variables (potentially) considered in the derivation of subgroup estimates
- Outcomes: outcomes measured and outcomes reported in the main analyses and subgroup analyses
- Declaration of interest and funding source

Extracted data will be compared and checked for accuracy; disagreements will be resolved through discussion and consensus.

#### Assessment of risk of bias in included studies

To assess the risk of bias of included studies, we will follow the guidance in the *Cochrane Handbook for Systematic Reviews of Interventions* [101]. Two review authors will independently assess the included studies considering also the main publications of the respective phase two and three studies using the Cochrane “Risk of Bias 2” tool (RoB2) for randomised trials [103], which will be implemented using the Rob2 Excel tool (https://sites.google.com/site/riskofbiastool/welcome/rob-2-0-tool/current-version-of-rob-2). Assessments will be conducted for each outcome included in the “Summary of findings” table. We will resolve disagreements in the risk of bias assessments by discussion and/or through consensus with a third author; if not possible, study authors will be contacted for clarification. The review is concerned with the effect of assignment to the interventions at baseline and will therefore follow the intention-to-treat (ITT) principle.

RoB2 includes five domains: (1) bias arising from the randomisation process, (2) bias due to deviations from the intended interventions, (3) bias due to missing outcome data, (4) bias in the measurement of the outcome and (5) bias in the selection of the reported result. Within each domain, a series of “signalling questions”, having the response options: “yes”, “probably yes”, “probably no”, “no” and “no information”, will be used to elicit information about trial features relevant to the risk of bias of a particular outcome. Based on the responses to the signalling questions, risk of bias judgement (i.e. “low risk of bias”, “some concerns” or “high risk of bias”) will be made for each of the five domains. Finally, using the domain-level judgements, we will reach one of the following three overall risk of bias judgements for a specific outcome of a trial:

1. “Low risk of bias”, if the trial was judged as low risk of bias in all five domains considered for the outcome.
2. “Some concerns”, if the trial was judged to raise some concerns in at least one domain for the outcome, but not at high risk of bias for any domain.
3. “High risk of bias”, if the trial was judged to be at high risk of bias in at least one domain for the outcome, or if it was judged to raise some concerns for multiple domains.

#### Measures of treatment effect

Treatment effects on rates (e.g. relapses, AE) will be quantified using rate ratios (RRs) when event rates are reported or computable, or using odds ratios (OR) when only proportions of affected patients are reported. Treatment effects on disability worsening will be quantified in terms of OR, based on the proportions of patients experiencing a sustained or confirmed disability worsening. Treatment effects on binary endpoints (e.g. disability worsening, NEDA) will be quantified in terms of OR, based on the proportions of affected patients. Treatment effects expressed in terms of rating scales (e.g. quality of life, fatigue) will be analysed based on standardised mean differences (SMD). Treatment effects expressed in terms of continuous endpoints (e.g. time to conversion) will be analysed based on mean differences. We will focus on differences in treatment effects between subgroups (technically an interaction effect of treatment and subgroup). Interpretation of SMD values will be based on the categories suggested by Cohen 1988 [104] (i.e., 0.2=”small”, 0.5=”medium”, 0.8=”large”). Estimates of overall mean effects will be quoted along with 95% confidence intervals (CI).

#### Unit of analysis issues

In the case of crossover trials, we will use only data from the first (pre-crossover) period. We will treat studies with more than two arms that would contribute to a joint analysis by adjusting standard errors to mimic a splitting of the placebo group into independent groups. When trials include several arms using the same DMT but with different modes of administration or different doses, we will consider only the arm with the approved dose or mode of administration.

#### Dealing with missing data

In case results are reported both in terms of ITT and per-protocol analysis sets, the ITT results will be preferred. Care will be taken to track the sample size corresponding to quoted estimates to be able to correctly quantify uncertainties. In case of missing or inadequate or inconsistent trial-level data, we will contact the principal investigator and primary authors to obtain additional information.

#### Assessment of heterogeneity

Between-trial heterogeneity will be accounted for by using random-effects models, where the heterogeneity parameter (the standard deviation tau) is estimated using the Paule-Mandel method [105]. We will judge heterogeneity (tau) on its absolute scale. In case there is evidence for the presence of “fairly extreme” heterogeneity [106], considering the heterogeneity estimate and its 95% CI, we will refrain from a quantitative synthesis and report a qualitative summary only.

#### Assessment of reporting biases

We will investigate reporting bias visually using funnel plots if we identify at least ten clinically and methodologically homogeneous studies that would contribute to a meta-analysis for the critical outcomes (concerning the “main analyses” described under the Data Synthesis section). If these visual assessments suggest asymmetry, we will use formal tests as suggested by Sterne and colleagues [103].

#### Data synthesis

If studies are sufficiently similar concerning participants, eligibility criteria and outcomes, we will pool effect estimates from all DMTs for the overall trial population with placebo as a control and conduct meta-analyses for each critical and important outcome. Following the procedure for the overall trial population, we will pool effect estimates from all DMTs, but this time for each subgroup with placebo as a control and conduct meta-analyses. We will then look into individual DMTs, at first with placebo as control and next with active controls. This analysis will precede the main analyses and illustrate the (combined) interventions’ overall effects.

Subsequently, we will address the main objectives. In particular, we will pool differences in effect estimates between the subgroup categories (e.g. effect differences between the categories “male” and “female” in the subgroup “sex”). In a second step, we will look into individual DMTs, at first with placebo as control and next with active controls (i.e. other approved DMTs), whereas placebo and active controls will be included in separate analyses. In case subgroups have more than two categories, we will dichotomise them. If not possible, we will conduct more than one analysis, based on reasonable summaries and integrations of reported subgroup categories. If subgroup category thresholds used in the identified studies are not alike, but similar enough to be reasonably combined, we will summarise respective studies (e.g. combine age subgroup categories of <38 (vs. >38) years and <40 (vs. >40) years into a younger (vs. older) group). If studies used different thresholds and thus cannot be summarised as described below, we will summarise studies based on the most frequently used thresholds for a particular subgroup.

We will summarise data for the following subgroups:

- Age: Baseline age will be dichotomised into a younger (<40 years) and older group (>40 years).
- Sex: Sex will be dichotomised into female and male groups.
- Treatment history: We will dichotomise treatment history into a treatment-naive group and a group in which participants had been treated with DMT(s) before entering the study (regardless of timeframe and type of DMT).
- Relapse rate: We will dichotomise participants based on the number of relapses per year before randomisation into a group with <1 relapse and a group with >2 or more relapses. In case the number of relapses in the previous 2 years are reported, these will be annualised by assuming a uniform distribution (e.g. 2 relapses in 24 months will be considered 1 relapse per year). Relapses will be defined as described under Types of outcome measures.
- Disability level: Based on baseline EDSS values, we will dichotomise participants into a group with low disability (EDSS <3.5) and a group with higher levels of disability (EDSS >3.5). Based on baseline MSFC scores, we will dichotomise participants into a group with below median scores and a group with equal or above median scores.
- MRI activity: We will dichotomise participants based on the number of baseline T2 hypointense lesions into a group with <9 lesions and a group with >9 T2 lesions and based on baseline T2 lesion volume into a group with below median (or mean) lesion volume and a group with equal or above median (or mean) lesion volume. We will dichotomise participants based on the number of contrast-enhancing T1 hyperintense lesions into a group without lesions and a group with >1 lesions, and based on baseline contrast-enhancing T1 hyperintense lesion volume into a group with below median lesion volume and a group with equal or above median lesion volume. We will dichotomise participants based on baseline lesion location into a group with infratentorial lesions and a group with supratentorial lesions.

Analyses will be carried out using random-effects models based on a normal-normal hierarchical model, combining effect (or interaction) estimates using generic inverse-variance methods and Paule-Mandel estimates for the heterogeneity variance. We will use “R” and the “metafor” packages [107–109].

In case a quantitative meta-analysis is not possible, we will attempt to summarise effect estimates and present a structured tabulation of the results, with outcomes ordered by e.g. risk of bias, according to the guidelines given in Chapter 12 of the *Cochrane Handbook for Systematic Reviews of Interventions* [107]. The main analyses of this review will include all eligible studies and will be concerned with outcomes reported 2 years post-randomisation.

#### Subgroup analysis and investigation of heterogeneity

If we identify a sufficient number of studies, we will conduct the following subgroup analyses:

- Different subtypes of MS, i.e. CIS or RRMS;
- Different diagnostic criteria, e.g. Poser, original McDonald or modified McDonald criteria
- DMTs grouped by level of efficacy, e.g. low efficacy DMTs (i.e. interferon-beta, glatiramer acetate, teriflunomide, dimethyl fumarate), moderate efficacy DMTs (i.e. cladribine, fingolimod, ozanimod) or high efficacy DMTs (i.e. ocrelizumab, mitoxantrone, alemtuzumab, natalizumab)
- Different definitions of disability worsening, e.g. increase in the EDSS of at least 1 point from baseline sustained for at least 3 or 6 months over a relapse-free period for baseline scores 5.5 or less, or of at least 0.5 points for baseline scores of more than 5.5
- Different follow-up periods, e.g. < 2 years or ≥ 2 years.

#### Sensitivity analysis

We will re-run the analyses (as described in the Data Synthesis section) excluding studies judged to be at high risk of bias in RoB2.

#### Summary of findings and assessment of the certainty of the evidence

We will assess the certainty of the evidence for the critical outcomes (concerning the "main analyses" described under Data synthesis) using the GRADE approach, which considers issues related to both internal validity (risk of bias, inconsistency, imprecision, publication bias) and external validity (directness of the results). Two review authors will independently rate the quality of the evidence for each outcome as “high”, “moderate”, “low” or “very low”; discrepancies (see above). The main results of this review will be presented in one or more “Summary of findings” table(s) in a transparent tabular format. For the application of the GRADE system and preparation of the “Summary of findings” table(s), we will follow the recommendations provided in Chapter 14 in the Cochrane Handbook for Systematic Reviews of Interventions [110].

## Discussion

A wealth of clinical trials has been conducted over the past decades in relapsing MS [111, 112]. For many RCTs investigating the efficacy of DMTs, sub-group analyses were conducted to identify groups of patients with larger treatment efficacy. While most individual trials found differences in the treatment effects for the examined subgroups, effects were mostly relatively small and did not challenge the overall trial's findings (i.e. DMTs were significantly more efficacious than placebo in almost all subgroups). While subgroup analyses of RCTs thus were, for most baseline factors considered, unable to identify TEM, this may be because clinical trials are rarely sufficiently powered to detect subgroup-specific effects [113]. Combining subgroup data from different studies in a meta-analysis could therefore identify patient groups consistently showing smaller/larger effects, due to the higher power of the meta-analytic approach. Despite the large number of RCTs conducted, the respective empirical evidence from subgroup analyses of clinical trials investigating DMT efficacy in people with RRMS has not been comprehensively and systematically assessed and summarised to date [51]. Evidence-based information on factors predicting significant differential treatment effects in people with RRMS are lacking for most approved DMTs and for people with highly active MS.

A tailored treatment approach that accounts for individuals’ characteristics and preferences, environmental aspects, disease features and biomolecular traits, will be particularly beneficial to improve and maximise outcomes in persons with MS [72, 114]. Solid knowledge about effect modification is therefore an essential element in personalised treatment algorithms [115].

There are some limitations to our planned review. We do not provide an extensive search to identify grey literature and might potentially miss some relevant studies. However, we will perform forward and backward reference screening and perform an extensive search in all relevant databases. Also, as we have no individual patient data access, this review will depend on the quality and comparability of the reported subgroup analyses.

We plan to publish the review in a peer-reviewed journal with an open access option.

## Supplementary Information

**Additional file 1.** Search strings.

## Data Availability

Not applicable

## Abbreviations

ADLs: Activities of daily living
AE: Adverse events
ARR: Annualised relapse rate
CI: Confidence interval
CIS: Clinically isolated syndrome
CNS: Central nervous system
DMT: Disease-modifying therapy
EDSS: Expanded Disability Status Scale
EMA: European Medicines Agency
EQ-5D: EuroQol-5 Dimension
FAMS: Functional Assessment of Multiple Sclerosis
FDA: U.S. Food and Drugs
FFS: Fatigue Severity Scale
FIM: Functional Independence Measure
HAQUAMS: Hamburg Quality of Life Questionnaire in Multiple Sclerosis
ITT: Intention to treat
JC: John Cunningham virus
LMSQoL: Leeds Multiple Sclerosis Quality of Life
MFI: Multidimensional Fatigue Index
MFIS: Modified Fatigue Impact Scale
MRI: Magnetic resonance imaging
MS: Multiple sclerosis
MSFC: Multiple sclerosis functional composite
MSQoL-54: Multiple Sclerosis Quality of Life-54
MUSIQoL: Multiple Sclerosis International Quality of Life questionnaire
NEDA: No Evidence of Disease Activity
OR: Odds ratio
PML: Progressive multifocal leukoencephalopathy
QoL: Quality of life
RCT: Randomised controlled trial
RRMS: Relapsing-remitting MS
RR: Rate ratio
SDM: Shared decision-making
SF-36: Health Status Questionnaire 36
SMD: Standardised mean difference
SPMS: Secondary progressive multiple sclerosis
TEM: Treatment effect modifier
VAS: Visual analogue scale for fatigue
