# Supplementary material for: Immunotherapy for people with clinically isolated syndrome or relapsing-remitting multiple sclerosis: treatment response by demographic, clinical, and biomarker subgroups (PROMISE)—a systematic review protocol
Source: Syst Rev. 2022 Jul 1;11:134. doi: 10.1186/s13643-022-01997-2 (PMC9250266; doi:10.1186/s13643-022-01997-2)
Supplement: Supplementary file 1 — Additional file 1. Search strings. [file 13643_2022_1997_MOESM1_ESM.docx]

**Appendix 1 search strings**

**MEDLINE (via Pubmed) (1946 to date)**

((((("Multiple Sclerosis"[Mesh:NoExp]) OR "Multiple Sclerosis, Relapsing-Remitting"[Mesh]) OR "Demyelinating Diseases"[Mesh:NoExp]) OR "Optic Neuritis"[Mesh]) OR "Demyelinating Autoimmune Diseases, CNS"[Mesh:NoExp]) OR "Encephalomyelitis, Acute Disseminated"[Mesh] OR "Multiple sclerosis"[Title/Abstract] OR "Disseminated Sclerosis"[Title/Abstract] OR "MS (Multiple Sclerosis)"[Title/Abstract] OR "Multiple Sclerosis, Acute Fulminating" [Title/Abstract] OR "Optic Neuritis"[Title/Abstract] OR "Optic Neuritides"[Title/Abstract] OR "devic disease"[Title/Abstract] OR "Remitting-Relapsing Multiple Sclerosis"[Title/Abstract] OR "Remitting Relapsing Multiple Sclerosis"[Title/Abstract] OR "Relapsing-Remitting Multiple Sclerosis"[Title/Abstract] OR "Relapsing Remitting Multiple Sclerosis"[Title/Abstract] OR "Acute Relapsing Multiple Sclerosis"[Title/Abstract] OR "Demyelinating Disease"[Title/Abstract] OR "Demyelinating Disorders"[Title/Abstract] OR "Demyelinating Disorder"[Title/Abstract] OR Demyelination[Title/Abstract] OR Demyelinations[Title/Abstract] OR "Clinically Isolated Syndrome, CNS Demyelinating"[Title/Abstract] OR "Clinically Isolated CNS Demyelinating Syndrome"[Title/Abstract] OR "clinically isolated syndrome"[Title/Abstract] OR "clinically isolated syndromes"[Title/Abstract] OR "clinically isolated syndrome CIS"[Title/Abstract] OR "first demyelinating event"[Title/Abstract] OR "first demyelinating events"[Title/Abstract] OR "first demyelinating episode"[Title/Abstract] OR "first demyelinating attack"[Title/Abstract] OR "First event"[Title/Abstract] OR "first episode"[Title/Abstract] OR "first clinical episode"[Title/Abstract] OR "single clinical episodes"[Title/Abstract] OR "first demyelinating event"[Title/Abstract] OR "first demyelinating events"[Title/Abstract] OR "clinically isolated syndrome"[Title/Abstract] OR "clinically isolated syndromes"[Title/Abstract] OR "Early-phase multiple sclerosis"[Title/Abstract] OR "Early phase multiple sclerosis"[Title/Abstract] OR “single clinical episode”[Title/Abstract] OR "transverse myelitis"[Title/Abstract] OR "acute disseminated encephalomyelitis"[Title/Abstract] OR "encephalomyelitis"[Title/Abstract] AND ((((((((((((("Alemtuzumab"[Mesh]) OR "Azathioprine"[Mesh]) OR "Cladribine"[Mesh]) OR "Dimethyl Fumarate"[Mesh]) OR "Fingolimod Hydrochloride"[Mesh]) OR "Glatiramer Acetate"[Mesh]) OR "Interferon beta-1a"[Mesh]) OR "Interferon beta-1b"[Mesh]) OR "Mitoxantrone"[Mesh]) OR "Natalizumab"[Mesh]) OR "ozanimod" [Supplementary Concept]) OR "ocrelizumab" [Supplementary Concept]) OR "siponimod" [Supplementary Concept]) OR "teriflunomide" [Supplementary Concept] OR Alemtuzumab[Title/Abstract] OR Campath[Title/Abstract] OR "Monoclonal Antibody Campath-1H"[Title/Abstract] OR MabCambath[Title/Abstract] OR Lemtrada[Title/Abstract] OR Lemtrada®[Title/Abstract] OR Azathioprine[Title/Abstract] OR Imurek®[Title/Abstract] OR Imuran®[Title/Abstract] OR Azothioprine[Title/Abstract] OR Imurel[Title/Abstract] OR Imuran[Title/Abstract] OR Immuran[Title/Abstract] OR Cladribine[Title/Abstract] OR Mavenclad®[Title/Abstract] OR Movectro®[Title/Abstract] OR Leustatin[Title/Abstract] OR "Dimethyl fumarate"[Title/Abstract] OR Tecfidera®[Title/Abstract] OR Dimethylfumarate[Title/Abstract] OR Tecfidera[Title/Abstract] OR Fumaderm[Title/Abstract] OR Fingolimod[Title/Abstract] OR Gilenya®[Title/Abstract] OR Gilenya[Title/Abstract] OR Gilenia[Title/Abstract] OR "Fingolimod Hydrochloride"[Title/Abstract] OR "Glatiramer acetate"[Title/Abstract] OR Copaxone®[Title/Abstract] OR Copaxone[Title/Abstract] OR Glatiramer[Title/Abstract] OR "Interferon beta-1a"[Title/Abstract] OR Rebif®[Title/Abstract] OR Avonex®[Title/Abstract] OR "Interferon beta 1a"[Title/Abstract] OR "Avonex Pen"[Title/Abstract] OR Rebif[Title/Abstract] OR Avonex[Title/Abstract] OR "interferon beta-1b"[Title/Abstract] OR Betaferon®[Title/Abstract] OR Betaseron®[Title/Abstract] OR Extavia®[Title/Abstract] OR "Beta-IFN-1b"[Title/Abstract] OR "Ser(17) IFN-beta"[Title/Abstract] OR "Serine(17) Interferon Beta"[Title/Abstract] OR "Interferon beta 1b"[Title/Abstract] OR "IFN-Beta Ser"[Title/Abstract] OR Betaseron[Title/Abstract] OR "Beta-Seron"[Title/Abstract] OR "Beta Seron"[Title/Abstract] OR Extavia[Title/Abstract] OR Betaferon[Title/Abstract] OR mitoxantrone[Title/Abstract] OR Novantron®[Title/Abstract] OR Ralenova®[Title/Abstract] OR Mitozantrone[Title/Abstract] OR Mitroxone[Title/Abstract] OR Pralifan[Title/Abstract] OR "Mitoxantrone Acetate"[Title/Abstract] OR "Mitoxantrone Hydrochloride"[Title/Abstract] OR Novantrone[Title/Abstract] OR Ralenova[Title/Abstract] OR Novantron[Title/Abstract] OR Onkotrone[Title/Abstract] OR natalizumab[Title/Abstract] OR Tysabri®[Title/Abstract] OR Tysabri[Title/Abstract] OR Antegren[Title/Abstract] OR ozanimod [Title/Abstract] OR Zeposia®[Title/Abstract] OR Ocrelizumab[Title/Abstract] OR Ocrevus®[Title/Abstract] OR Ocrevus[Title/Abstract] OR Siponimod [Title/Abstract] OR Mayzent®[Title/Abstract] OR Mayzent[Title/Abstract] OR Teriflunomide [Title/Abstract] OR Aubagio®[Title/Abstract] OR Ofatumumab[Title/Abstract] OR Kesimpta®[Title/Abstract] OR arzerra[Title/Abstract] AND ((((((((("randomized controlled trial"[Publication Type] OR "controlled clinical trial"[Publication Type]) OR "randomized"[Title/Abstract]) OR "placebo"[Title/Abstract]) OR "clinical trials as topic"[MeSH Terms:noexp]) OR "randomly"[Title/Abstract]) OR "trial"[Title]) OR "subgroup*"[Title/Abstract]) OR ("animals"[MeSH Terms] NOT "humans"[MeSH Terms]))

**Embase (1974 to date)**

#1 'multiple sclerosis'/exp/mj OR 'demyelinating disease'/exp/mj OR 'optic neuritis'/exp/mj OR 'acute disseminated encephalomyelitis'/exp/mj

#2 'multiple sclerosis' OR 'disseminated sclerosis' OR 'ms (multiple sclerosis)' OR 'multiple sclerosis, acute fulminating' OR 'optic neuritis' OR 'optic neuritides' OR 'devic disease' OR 'remitting-relapsing multiple sclerosis' OR 'remitting relapsing multiple sclerosis' OR 'relapsing-remitting multiple sclerosis' OR 'relapsing remitting multiple sclerosis' OR 'acute relapsing multiple sclerosis' OR 'demyelinating disease' OR 'demyelinating disorders' OR 'demyelinating disorder' OR demyelination OR demyelinations OR 'clinically isolated syndrome, cns demyelinating' OR 'clinically isolated cns demyelinating syndrome' OR 'clinically isolated syndrome cis' OR 'first demyelinating episode' OR 'first demyelinating attack' OR 'first event' OR 'first episode' OR 'first clinical episode' OR 'single clinical episodes' OR 'first demyelinating event' OR 'first demyelinating events' OR 'clinically isolated syndrome' OR 'clinically isolated syndromes' OR 'early-phase multiple sclerosis' OR 'early phase multiple sclerosis' OR 'single clinical episode' OR 'transverse myelitis' OR 'acute disseminated encephalomyelitis' OR 'encephalomyelitis':ti,ab

#3 #1 OR #2

#4 'alemtuzumab'/exp/mj OR 'azathioprine'/exp/mj OR 'cladribine'/exp/mj OR 'fumaric acid dimethyl ester'/exp/mj OR 'fingolimod'/exp/mj OR 'glatiramer'/exp/mj OR 'beta1a interferon'/exp/mj OR 'interferon beta serine'/exp/mj OR 'mitoxantrone'/exp/mj OR 'natalizumab'/exp/mj OR 'ocrelizumab'/exp/mj OR 'siponimod'/exp/mj OR 'teriflunomide'/exp/mj OR 'ofatumumab'/exp/mj

#5 (alemtuzumab OR campath OR 'monoclonal antibody campath-1h' OR mabcambath OR lemtrada OR lemtrada® OR azathioprine OR imurek® OR imuran® OR azothioprine OR imurel OR imuran OR immuran OR cladribine OR mavenclad® OR movectro® OR leustatin OR 'dimethyl fumarate' OR tecfidera® OR dimethylfumarate OR tecfidera OR fumaderm OR fingolimod OR gilenya® OR gilenya OR gilenia OR 'fingolimod hydrochloride' OR 'glatiramer acetate' OR copaxone® OR copaxone OR glatiramer OR 'interferon beta-1a' OR rebif® OR avonex® OR 'interferon beta 1a' OR 'avonex pen' OR rebif OR avonex OR 'interferon beta-1b' OR betaferon® OR betaseron® OR extavia® OR 'beta-ifn-1b' OR 'ser(17) ifn-beta' OR 'serine(17) interferon beta' OR 'interferon beta 1b' OR 'ifn-beta ser' OR betaseron OR 'beta-seron' OR 'beta seron' OR extavia OR betaferon OR mitoxantrone OR novantron® OR ralenova® OR mitozantrone OR mitroxone OR pralifan OR 'mitoxantrone acetate' OR 'mitoxantrone hydrochloride' OR novantrone OR ralenova OR novantron OR onkotrone OR natalizumab OR tysabri® OR tysabri OR antegrenor) AND ozanimod OR zeposia® OR ocrelizumab OR ocrevus® OR ocrevus OR siponimod OR mayzent® OR mayzent OR teriflunomide OR aubagio® OR 'ofatumumab' OR Kesimpta® OR arzerra:ti,ab

#6 #4 OR #5

#7 'crossover procedure':de OR 'double-blind procedure':de OR 'randomized controlled trial':de OR 'single-blind procedure':de OR random*:de,ab,ti OR factorial*:de,ab,ti OR crossover*:de,ab,ti OR ((cross NEXT/1 over*):de,ab,ti) OR placebo*:de,ab,ti OR ((doubl* NEAR/1 blind*):de,ab,ti) OR ((singl* NEAR/1 blind*):de,ab,ti) OR assign*:de,ab,ti OR allocat*:de,ab,ti OR volunteer*:de,ab,ti OR subgroup*:de,ab,ti

#8 #3 AND #6 AND #7

**Cochrane Central Register of Controlled Trials (CENTRAL; 2020, most recent issue) in the Cochrane Library**

#1 MeSH descriptor: [Multiple Sclerosis] this term only

#2 MeSH descriptor: [Multiple Sclerosis, Relapsing-Remitting] this term only

#3 MeSH descriptor: [Demyelinating Autoimmune Diseases, CNS] 1 tree(s) exploded

#4 MeSH descriptor: [Optic Neuritis] this term only

#5 MeSH descriptor: [Encephalomyelitis, Acute Disseminated] explode all trees

#6 ("Multiple sclerosis" OR "Disseminated Sclerosis" OR "MS (Multiple Sclerosis)" OR "Multiple Sclerosis, Acute Fulminating" OR "Optic Neuritis" OR "Optic Neuritides" OR "devic disease" OR "Remitting-Relapsing Multiple Sclerosis" OR "Remitting Relapsing Multiple Sclerosis" OR "Relapsing-Remitting Multiple Sclerosis" OR "Relapsing Remitting Multiple Sclerosis" OR "Acute Relapsing Multiple Sclerosis" OR "Demyelinating Disease" OR "Demyelinating Disorders" OR "Demyelinating Disorder" OR Demyelination OR Demyelinations OR "Clinically Isolated Syndrome, CNS Demyelinating" OR "Clinically Isolated CNS Demyelinating Syndrome" OR "clinically isolated syndrome" OR "clinically isolated syndromes" OR "clinically isolated syndrome CIS" OR "first demyelinating event" OR "first demyelinating events" OR "first demyelinating episode" OR "first demyelinating attack" OR "First event" OR "first episode" OR "first clinical episode" OR "single clinical episodes" OR "first demyelinating event" OR "first demyelinating events" OR "clinically isolated syndrome" OR "clinically isolated syndromes" OR "Early-phase multiple sclerosis" OR "Early phase multiple sclerosis" OR “single clinical episode” OR "transverse myelitis" OR "acute disseminated encephalomyelitis" OR "encephalomyelitis"):ti,ab,kw

#7 {OR #1-#6}

#8 MeSH descriptor: [Alemtuzumab] explode all trees

#9 MeSH descriptor: [Azathioprine] explode all trees

#10 MeSH descriptor: [Cladribine] explode all trees

#11 MeSH descriptor: [Dimethyl Fumarate] explode all trees

#12 MeSH descriptor: [Fingolimod Hydrochloride] explode all trees

#13 MeSH descriptor: [Glatiramer Acetate] explode all trees

#14 MeSH descriptor: [Interferon beta-1a] explode all trees

#15 MeSH descriptor: [Interferon beta-1b] explode all trees

#16 MeSH descriptor: [Mitoxantrone] explode all trees

#17 MeSH descriptor: [Natalizumab] explode all trees

#18 (Alemtuzumab OR Campath OR "Monoclonal Antibody Campath-1H" OR MabCambath OR Lemtrada OR Lemtrada® OR Azathioprine OR Imurek® OR Imuran® OR Azothioprine OR Imurel OR Imuran OR Immuran OR Cladribine OR Mavenclad® OR Movectro® OR Leustatin OR "Dimethyl fumarate" OR Tecfidera® OR ):ti,ab,kw

#19 (Dimethylfumarate OR Tecfidera OR Fumaderm OR Fingolimod OR Gilenya® OR Gilenya OR Gilenia OR "Fingolimod Hydrochloride" OR "Glatiramer acetate" OR Copaxone® OR Copaxone OR Glatiramer OR "Interferon beta-1a" OR Rebif® OR Avonex® OR "Interferon beta 1a"):ti,ab,kw

#20 ("Avonex Pen" OR Rebif OR Avonex OR "interferon beta-1b" OR Betaferon® OR Betaseron® OR Extavia® OR "Beta-IFN-1b" OR "Ser(17) IFN-beta" OR "Serine(17) Interferon Beta" OR "Interferon beta 1b" OR "IFN-Beta Ser" OR Betaseron OR "Beta-Seron" OR "Beta Seron" OR Extavia):ti,ab,kw

#21 (Betaferon OR mitoxantrone OR Novantron® OR Ralenova® OR Mitozantrone OR Mitroxone OR Pralifan OR "Mitoxantrone Acetate" OR "Mitoxantrone Hydrochloride" OR Novantrone OR Ralenova OR Novantron OR Onkotrone OR natalizumab OR Tysabri® OR Tysabri OR Antegren OR ozanimod OR Zeposia® OR Ocrelizumab OR Ocrevus® OR Ocrevus OR Siponimod OR Mayzent® OR Mayzent OR Teriflunomide OR Aubagio® OR 'ofatumumab' OR Kesimpta® OR arzerra):ti,ab,kw

#22 {OR #8-#21}

#23 #7 AND #22

**Cumulative Index to Nursing and Allied Health Literature (CINAHL) (EBSCOhost) (1981 to date)**

S1 MH Multiple Sclerosis OR MH Multiple Sclerosis, Relapsing-Remitting OR MH Demyelinating Autoimmune Diseases, CNS OR MH Optic Neuritis OR MH Encephalomyelitis, Acute Disseminated OR AB ( "Multiple sclerosis" OR "Disseminated Sclerosis" OR "MS (Multiple Sclerosis)" OR "Multiple Sclerosis, Acute Fulminating" OR "Optic Neuritis" OR "Optic Neuritides" OR "devic disease" OR "Remitting-Relapsing Multiple Sclerosis" OR "Remitting Relapsing Multiple Sclerosis" OR "Relapsing-Remitting Multiple Sclerosis" OR "Relapsing Remitting Multiple Sclerosis" OR "Acute Relapsing Multiple Sclerosis" OR "Demyelinating Disease" OR "Demyelinating Disorders" OR "Demyelinating Disorder" OR Demyelination OR Demyelinations OR "Clinically Isolated Syndrome, CNS Demyelinating" OR "Clinically Isolated CNS Demyelinating Syndrome" OR "clinically isolated syndrome" OR "clinically isolated syndromes" OR "clinically isolated syndrome CIS" OR "first demyelinating event" OR "first demyelinating events" OR "first demyelinating episode" OR "first demyelinating attack" OR "First event" OR "first episode" OR "first clinical episode" OR "single clinical episodes" OR "first demyelinating event" OR "first demyelinating events" OR "clinically isolated syndrome" OR "clinically isolated syndromes" OR "Early-phase multiple sclerosis" OR "Early phase multiple sclerosis" OR “single clinical episode” OR "transverse myelitis" OR "acute disseminated encephalomyelitis" OR "encephalomyelitis )

S2 MH Alemtuzumab OR MH Azathioprine OR MH Cladribine OR MH Dimethyl Fumarate OR MH Fingolimod Hydrochloride OR MH Glatiramer Acetate OR MH Interferon beta-1a OR MH Interferon beta-1b OR MH Mitoxantrone OR MH Natalizumab OR AB ( Alemtuzumab OR Campath OR "Monoclonal Antibody Campath-1H" OR MabCambath OR Lemtrada OR Lemtrada® OR Azathioprine OR Imurek® OR Imuran® OR Azothioprine OR Imurel OR Imuran OR Immuran OR Cladribine OR Mavenclad® OR Movectro® OR Leustatin OR "Dimethyl fumarate" OR Tecfidera® OR Dimethylfumarate OR Tecfidera OR Fumaderm OR Fingolimod OR Gilenya® OR Gilenya OR Gilenia OR "Fingolimod Hydrochloride" OR "Glatiramer acetate" OR Copaxone® OR Copaxone OR Glatiramer OR "Interferon beta-1a" OR Rebif® OR Avonex® OR "Interferon beta 1a" OR "Avonex Pen" OR Rebif OR Avonex OR "interferon beta-1b" OR Betaferon® OR Betaseron® OR Extavia® OR "Beta-IFN-1b" OR "Ser(17) IFN-beta" OR "Serine(17) Interferon Beta" OR "Interferon beta 1b" OR "IFN-Beta Ser" OR Betaseron OR "Beta-Seron" OR "Beta Seron" OR Extavia OR Betaferon OR mitoxantrone OR Novantron® OR Ralenova® OR Mitozantrone OR Mitroxone OR Pralifan OR "Mitoxantrone Acetate" OR "Mitoxantrone Hydrochloride" OR Novantrone OR Ralenova OR Novantron OR Onkotrone OR natalizumab OR Tysabri® OR Tysabri OR Antegren OR ozanimod OR Zeposia® OR Ocrelizumab OR Ocrevus® OR Ocrevus OR Siponimod OR Mayzent® OR Mayzent OR Teriflunomide OR Aubagio® OR 'ofatumumab' OR Kesimpta® OR arzerra)

S3 S1 AND S2

S4 MJ "randomized controlled trial" OR AB "randomized controlled trial" OR TI (randomized" OR "placebo" OR "randomly" OR "trial" OR "subgroup*")

S5 S3 AND S4

**Latin American and Caribbean Health Science Information Database (LILACS) (Bireme) (1982 to date)**

tw:((tw:("multiple sclerosis" OR "Relapsing-Remitting Multiple Sclerosis" OR "clinically isolated syndrome")) AND (tw:(Alemtuzumab OR Campath OR "Monoclonal Antibody Campath-1H" OR MabCambath OR Lemtrada OR Lemtrada® OR Azathioprine OR Imurek® OR Imuran® OR Azothioprine OR Imurel OR Imuran OR Immuran OR Cladribine OR Mavenclad® OR Movectro® OR Leustatin OR "Dimethyl fumarate" OR Tecfidera® OR Dimethylfumarate OR Tecfidera OR Fumaderm OR Fingolimod OR Gilenya® OR Gilenya OR Gilenia OR "Fingolimod Hydrochloride" OR "Glatiramer acetate" OR Copaxone® OR Copaxone OR Glatiramer OR "Interferon beta-1a" OR Rebif® OR Avonex® OR "Interferon beta 1a" OR "Avonex Pen" OR Rebif OR Avonex OR "interferon beta-1b" OR Betaferon® OR Betaseron® OR Extavia® OR "Beta-IFN-1b" OR "Ser(17) IFN-beta" OR "Serine(17) Interferon Beta" OR "Interferon beta 1b" OR "IFN-Beta Ser" OR Betaseron OR "Beta-Seron" OR "Beta Seron" OR Extavia OR Betaferon OR mitoxantrone OR Novantron® OR Ralenova® OR Mitozantrone OR Mitroxone OR Pralifan OR "Mitoxantrone Acetate" OR "Mitoxantrone Hydrochloride" OR Novantrone OR Ralenova OR Novantron OR Onkotrone OR natalizumab OR Tysabri® OR Tysabri OR Antegren OR ozanimod OR Zeposia® OR Ocrelizumab OR Ocrevus® OR Ocrevus OR Siponimod OR Mayzent® OR Mayzent OR Teriflunomide OR Aubagio® OR 'ofatumumab' OR Kesimpta® OR arzerra))
